# Evaluation of a Quantitative Serological Assay for Diagnosing Chronic Pulmonary Aspergillosis

**DOI:** 10.1128/JCM.01475-15

**Published:** 2016-05-23

**Authors:** Satoru Fujiuchi, Yuka Fujita, Hokuto Suzuki, Kazushi Doushita, Hikaru Kuroda, Masaaki Takahashi, Yasuhiro Yamazaki, Tadakatsu Tsuji, Toshiaki Fujikane, Shinobu Osanai, Takaaki Sasaki, Yoshinobu Ohsaki

**Affiliations:** a Department of Respiratory Medicine, National Hospital Organization, Asahikawa Medical Center, Asahikawa, Japan; b Cardiovascular Respiratory Frontier of Medical Renovation, Asahikawa Medical University, Asahikawa, Japan; c Respiratory Center, Asahikawa Medical University, Asahikawa, Japan

## Abstract

The purpose of this study was to evaluate the clinical utility of a quantitative Aspergillus IgG assay for diagnosing chronic pulmonary aspergillosis. We examined Aspergillus-specific IgG levels in patients who met the following criteria: (i) chronic (duration of >3 months) pulmonary or systemic symptoms, (ii) radiological evidence of a progressive (over months or years) pulmonary lesion with surrounding inflammation, and (iii) no major discernible immunocompromising factors. Anti-Aspergillus IgG serum levels were retrospectively analyzed according to defined classifications. Mean Aspergillus IgG levels were significantly higher in the proven group than those in the possible and control groups (*P* < 0.01). Receiver operating characteristic curve analysis revealed that the Aspergillus IgG cutoff value for diagnosing proven cases was 50 mg of antigen-specific antibodies/liter (area under the curve, 0.94; sensitivity, 0.98; specificity, 0.84). The sensitivity and specificity for diagnosing proven cases using this cutoff were 0.77 and 0.78, respectively. The positive rates of Aspergillus IgG in the proven and possible groups were 97.9% and 39.2%, respectively, whereas that of the control group was 6.6%. The quantitative Aspergillus IgG assay offers reliable sensitivity and specificity for diagnosing chronic pulmonary aspergillosis and may be an alternative to the conventional precipitin test.

## INTRODUCTION

Chronic pulmonary aspergillosis (CPA) usually occurs in patients with underlying pulmonary disease (1), and the lesion usually progresses latently. Therefore, it is not uncommon for patients to suddenly develop hemoptysis and/or respiratory failure. CPA is considered to be one of the most refractory pulmonary infectious diseases; the estimated 5-year survival rate of CPA is 50%, which is similar to that of idiopathic pulmonary fibrosis (2). Because Aspergillus is the causative agent, isolation of the responsible Aspergillus species from the airway tract is important for diagnosis, but the rate of isolation on sputum culture examination is relatively low (3). At present, serum detection of IgG antibodies to Aspergillus is considered to be the most reliable method for diagnosing CPA (4). Immunodiffusion analysis is widely used to detect the anti-Aspergillus antibody precipitin; however, it takes up to 1 week to obtain results. Recent studies have described the use of fluorescent immunoenzyme assays for quantifying IgGs to Aspergillus and have reported that it seemed to be more sensitive than conventional anti-Aspergillus antibody detection (5, 6). In this study, we examined the utility of quantitative measurement of IgG to Aspergillus for diagnosing CPA.

(This study was presented in part at the European Respiratory Society International Congress, Munich, Germany, 2014.).

## MATERIALS AND METHODS

This retrospective study was performed at the National Hospital Organization Asahikawa Medical Center. From January 2007 to August 2013, 269 patients with underlying chronic respiratory disease who were considered to have CPA based on three criteria (Table 1) were examined. These criteria were (i) chronic (duration of >3 months) pulmonary or systemic symptoms (e.g., cough, bloody sputum, hemoptysis, pyrexia, or dyspnea), (ii) radiological evidence of a progressive (over months or years) pulmonary lesion with surrounding inflammation (e.g., cavitation, infiltration, and pleural thickening), and (iii) no major discernible immunocompromising factors (e.g., AIDS, leukemia, or transplantation). There were no patients treated with corticosteroids (more than a dose of 0.3 mg/kg of body weight per day), cyclosporine, tumor necrosis factor alpha (TNF-α) blockers, or specific monoclonal antibodies. Patients with a history of antifungal treatment that could affect antibody values were excluded. Cases of stable nontuberculous mycobacteriosis with negative sputum culture were also enrolled. Circulating anti-Aspergillus antibodies were examined using the immunodiffusion method. The residual serum was stored at −80°C for further analysis to measure the concentration of a specific IgG to Aspergillus. The patients were divided into three groups according to antibody status and inflammation markers. Patients with circulating precipitating (IgG) antibody to Aspergillus and persistently elevated inflammation markers (C-reactive protein of >0.3 mg/dl or white blood cell counts of >9,000/μl for >3 months) were defined as proven CPA. Patients without circulating precipitating (IgG) antibody to Aspergillus and persistently elevated inflammation markers were considered to have possible CPA. Patients with temporarily elevated inflammation markers with any circulating precipitating (IgG) antibody were defined as controls. The classification of proven CPA is based on the diagnostic criteria proposed by Denning et al. (7). We examined anti-Aspergillus precipitin levels with an Aspergillus immunodiffusion FSK-1 kit (Microgen Bioproducts Ltd., Camberley, United Kingdom) according to the manufacturer's instructions. Briefly, the immunodiffusion reaction was performed in agarose gel for 3 days. After stringent washing, the agarose gel was stained with crystal violet. Precipitin was considered positive if precipitation arcs were visible to 2 mg/ml for Aspergillus fumigatus somatic antigen and culture filtrate.

**TABLE 1:** Patient characteristics

Characteristic	Control (*n* = 122)	Possible (*n* = 51)	Proven (*n* = 96)	*P* value
Age, yr	71.9	75.4	7.35	ns^*a*^
No. male/female	74/48	42/9	78/18	0.01
No. with underlying pulmonary disease (%)				0.01
Sequelae of tuberculosis	28 (23.0)	13 (25.5)	37 (38.5)	
COPD^*b*^	23 (18.9)	14 (27.5)	22 (22.9)	
Pulmonary fibrosis	32 (26.2)	9 (17.6)	7 (7.3)	
Nontuberculous mycobacteriosis	11 (9.0)	5 (9.8)	13 (13.5)	
Bronchiectasis	19 (15.6)	8 (15.7)	4 (4.2)	
Bullae of lung	8 (6.5)	1 (1.9)	11 (11.5)	
Other	1 (0.8)	1 (1.9)	2 (2.1)	

a ns, Not significant.

b COPD, chronic obstructive pulmonary disease.

Quantification of specific IgG to Aspergillus in collected serum was performed using the ImmunoCAP method (Phadia, Uppsala, Sweden). We employed fluorescent immunoenzyme assays to measure the concentration of a specific IgG to Aspergillus using Gm3 ImmunoCap as an antigen. All statistic tests and receiver operating characteristic (ROC) curve analyses were performed with SPSS version 23.0 (IBM Corp., Armonk, NY, USA).

## RESULTS

**FIG 1:**
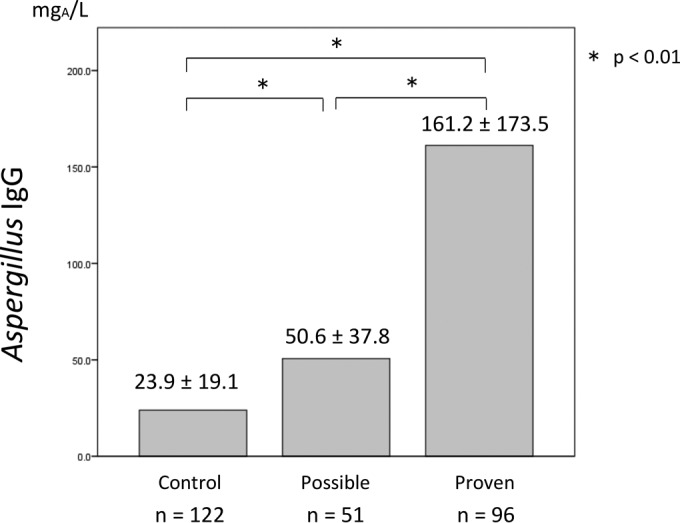
Average Aspergillus IgG level in serum from each group measured by fluorescent immunoenzyme assay. Data represent the means ± standard deviations (SDs). *, *P* value of <0.01.

**FIG 2:**
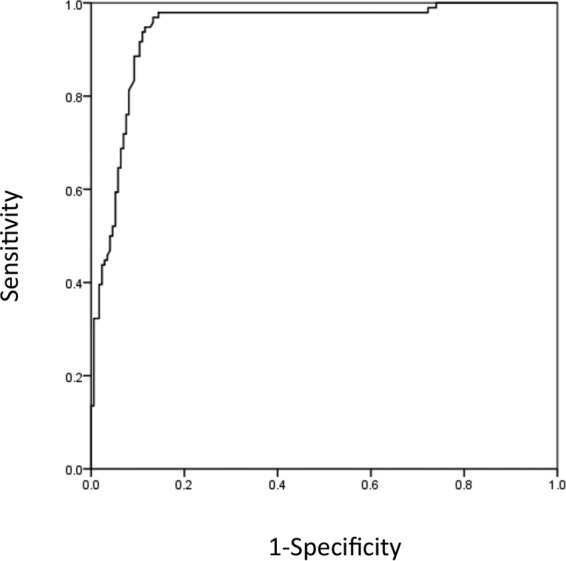
ROC analysis for diagnosing probable and proven cases. The optimal cutoff value of Aspergillus IgG for diagnosing proven and probable cases was 50 mg_A_/liter (AUC, 0.94; 95% confidence interval, 0.912 to 0.972; sensitivity, 0.98; specificity, 0.84).

**FIG 3:**
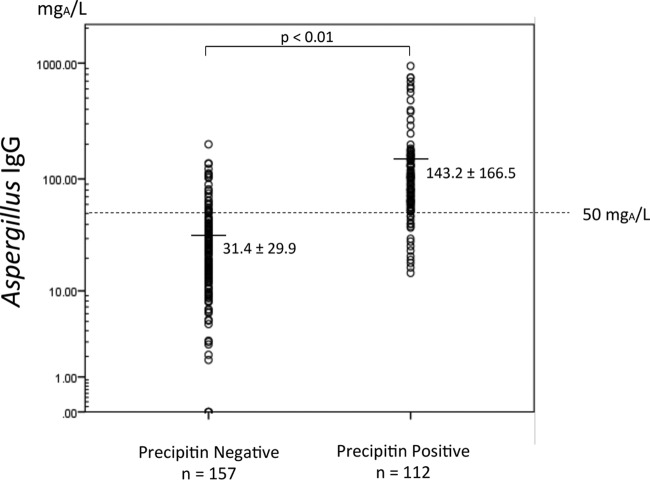
The comparison of anti-Aspergillus precipitin (IgG) with specific Aspergillus IgG levels. Specific IgG was significantly higher in precipitin-positive patients; however, there were some positive cases with low Aspergillus IgG levels.

The specific Aspergillus IgG level was significantly higher in the proven CPA group (161.2 mg of antigen-specific antibodies [mg_A_]/liter) than that in the control group (23.9 mg_A_/liter; *P* < 0.01) (Fig. 1). In the possible CPA group, the specific Aspergillus IgG level was 50.6 mg_A_/liter, which was also higher than that in the control group. ROC curve analysis revealed that the optimal Aspergillus IgG cutoff for diagnosing proven CPA was 50 mg_A_/liter (area under the curve [AUC], 0.94; 95% confidence interval, 0.912 to 0.972; sensitivity, 0.98; specificity, 0.84 at a cutoff of 50 mg_A_/liter) (Fig. 2). Whereas the current cutoff value (40 mg_A_/liter) indicated that sensitivity and specificity were 0.97 and 0.75, respectively. The cutoff value at 50 mg_A_/liter showed better sensitivity and specificity than those at 40 mg_A_/liter. The positive rates of Aspergillus IgG using this cutoff in the proven and possible groups were 97.9% and 39.2%, respectively, whereas the control group was 6.6% (Table 2). The sensitivity and specificity for diagnosing proven cases using a cutoff of 50 mg_A_/liter were 0.77 and 0.78, respectively. The comparison of anti-Aspergillus precipitin (IgG) with specific Aspergillus IgG concentration is shown in Fig. 3. Specific IgG was significantly higher in precipitin-positive patients; however, there were some cases with low Aspergillus IgG levels.

**TABLE 2:** Positive rates for the quantitative Aspergillus IgG assay (cutoff, 50 mg_A_/liter)

Result	No. positive (%)	No. negative (%)
Proven^*a*^	94 (97.9)	2 (2.1)
Possible^*b*^	20 (39.2)	31 (60.8)
Control^*c*^	8 (6.6)	114 (93.4)

a Aspergillus precipitin positive and a persistently elevated inflammation marker.

b Aspergillus precipitin negative and a persistently elevated inflammation marker.

c Any Aspergillus precipitin and temporary elevated inflammation marker.

## DISCUSSION

According to Japanese autopsy records, the frequency of deep mycoses gradually decreased to ∼3% by 1994, but it subsequently increased and reached 4.6% in 2001 (8). Although the prevalence of candidiasis continues to decrease and now accounts for only 40% of deep mycoses compared to that in 1990, the increase in aspergillosis persists and is currently considered a major fungal infection. Tashiro et al. reported increased isolation of Aspergillus spp. from respiratory specimens in Nagasaki, Japan (9). These epidemical alternations may be due to the increasing number of elderly subjects with pulmonary comorbidities in our country. Thus, the issue of Aspergillus infection may be focused not only in Japan but also in other developed nations.

The quality of life of CPA patients is drastically reduced in the advanced stages of disease. Although CPA lesions progress silently, the 5-year survival rate of CPA patients is estimated to be around 50% (2, 10, 11). We previously reported that the number of lesions found in the lobes of the lungs predicts the prognosis (12). Therefore, early diagnosis and timely intervention may improve CPA patient prognosis. The detection of pathogenic fungi is the basis of infectious disease diagnosis, but because Aspergillus species are ubiquitous, their isolation from the respiratory tract is not always evidence of pathology. Only surgical resection and pathology findings can provide a definitive diagnosis. However, such procedures are not usually possible because of the patients' poor general condition.

It is important to carry out serodiagnostic examinations in early-stage CPA. Screening examinations should be performed when any of the following occur: a new infiltrative shadow, hollow expansion, hyperplasia of a cavity wall, progressive pleural thickening, niveau formation on a chest X-ray in patients with old tuberculosis, pulmonary cyst, pulmonary fibrosis, or chronic obstructive pulmonary disease. Measuring galactomannan antigen levels is not useful for CPA diagnosis because of its low sensitivity (13). Denning et al. proposed a set of diagnostic criteria in combination with clinical and anti-Aspergillus antibody conditions (7). To date, detection of the precipitating anti-Aspergillus antibody is considered the most reliable method and is used for CPA diagnosis in Japan.

Three types of anti-Aspergillus antibody detection techniques are accepted worldwide. Baxter et al. reported that the IgG quantification assay using a fluorescent immunoenzyme assay (ImmunoCAP) is superior to conventional precipitin detection (6), and this method recently became available in Japan (5). In the present study, we examined the utility of quantitative measurement of IgG to Aspergillus for diagnosing CPA. To detect anti-Aspergillus precipitin, we used the same antigen that Baxter et al. employed in counterimmunoelectrophoresis (6). In contrast to their study, we analyzed subjects who had not been treated with antifungals. We found that Aspergillus IgG levels were significantly higher in the proven group than those in the control group. The current cutoff value (40 mg_A_/liter) was decided by United Kingdom laboratories following a consensus meeting between six United Kingdom laboratories and Phadia. To our knowledge, there is no published exploratory study for an optimal cutoff value for untreated CPA, so identifying one was a goal of the present work. ROC analysis showed that the best sensitivity and specificity for proven cases were at a cutoff of 50 mg_A_/liter, which was relatively higher than the usual cutoff value (40 mg_A_/liter). It is reported that allergic bronchopulmonary aspergillosis (ABPA) patients exhibit rapidly lower anti-Aspergillus IgG levels after antifungal treatment. We excluded ABPA patients and antifungal-treated subjects, which may have influenced the higher cutoff value. Still, the sensitivity and specificity for proven cases were 0.77 and 0.78, respectively. Furthermore, >95% of proven cases had levels of >50 mg_A_/liter. It is thought that this cutoff is optimal for untreated subjects. Our results confirm that measuring specific IgGs to Aspergillus is useful for diagnosing CPA. However, we should mention that it is not clear why a few precipitin-positive cases had Aspergillus IgG levels of <50 mg_A_/liter (Table 3). Further analyses are required to explain this discrepant finding.

**TABLE 3:** Detection power of the quantitative Aspergillus IgG assay

Precipitin result	No. at >50 mg_A_/liter	No. at <49.9 mg_A_/liter
Positive	96	16
Negative	26	131

In the clinical setting, physicians often encounter patients who are suspected to have CPA based on their clinical course. It is important to determine whether specific IgG levels are elevated in these “gray” cases. Interestingly, anti-Aspergillus antibody levels in the possible CPA group were higher than those in the control group. In addition, subsequent precipitin tests were positive in 4 out of 50 possible CPA cases. This suggests that the specific IgG test might have been more sensitive than precipitin detection. Therefore, earlier intervention may improve the prognosis of CPA.

Because of the retrospective single facility study, the limitation of patient selection bias is a concern. However, our findings and the findings of others demonstrate the clinical utility of measuring specific IgG for Aspergillus. Furthermore, IgG quantification might be useful for monitoring antifungal treatment responses. The matter of changing of anti-Aspergillus IgG in relation to clinical course or treatment should be analyzed in the future. In conclusion, the Aspergillus IgG fluorescent immunoenzyme assay is a reliable test for diagnosing CPA and may be a suitable replacement for conventional precipitin tests.
